# Fungal-fermented corn straw as an organic amendment: balancing tomato nutrition, soil functions and antibiotic resistance

**DOI:** 10.3389/fpls.2026.1765584

**Published:** 2026-02-27

**Authors:** Tianhao Song, Xiaoyan Zhou, Yongsheng Ma, Longfei Chen, Xiulian Duan, Yueting Dai

**Affiliations:** Engineering Research Center of Edible and Medicinal Fungi, Ministry of Education, Jilin Agricultural University, Changchun, China

**Keywords:** ARGS, FSP, microbial community, soil functions/enzymes, tomato quality

## Abstract

**Introduction:**

Tomato growers need strategies that improve fruit nutritional quality and soil health while reducing dependence on synthetic fertilizers and wasting less crop straw. Fungal-fermented straw products (FSP) are a candidate amendment, but their suitable application rate, placement depth and side effects on soil microbes and antibiotic resistance genes (ARGs) remain unclear.

**Methods:**

An FSP was produced from corn straw via solid-state fermentation for 30 d using *Auricularia cornea* cv. Yumuer. A single-season greenhouse pot experiment was conducted with an unamended control (no FSP) and six FSP treatments combining different rates (0.5–5% w/w) and soil incorporation depths (3 and 10 cm). Measurements included tomato yield (growth and fruit yield), fruit quality, soil health, biochemical responses, and molecular responses.

**Results:**

FSP at 2% (w/w) incorporated to 10 cm increased yield by about 30% and raised fruit lycopene and vitamin C by 20–40% compared with the control. It also enhanced soil organic carbon, available P and K, humic substances and key enzymes, and shifted microbes toward decomposers and plant-beneficial taxa (e.g., *Streptomyces*, *Sphingomonas*, *Nocardioides*, and *Arthrobacter*). A high surface dose (5% at 3 cm) increased total ARGs, whereas 2% at 10 cm achieved quality and soil benefits with ARG levels comparable to or lower than the control.

**Discussion:**

These results suggest a practical application pattern that balances agronomic benefits with ARG-related risk for recycling crop residues in intensive tomato systems.

## 1 Introduction

Tomatoes (*Solanum lycopersicum*) are among the most widely consumed vegetables worldwide (Włodarczyk et al., 2022), valued not only for their yield but also for their nutritional and flavor attributes. Tomato fruits provide health-promoting compounds such as lycopene and vitamin C, which function as antioxidants (Haraira et al., 2022). With consumers increasingly seeking produce that combines high nutritional value and appealing taste, research priorities have shifted toward enhancing fruit quality traits (e.g., soluble sugars, vitamins, phytochemicals) alongside yield. Conventional intensive farming practices rely heavily on chemical fertilizers (Montgomery and Biklé, 2021), which ensure high yields but may compromise nutritional and organoleptic qualities. Sustainable alternatives, such as organic amendments and biofertilizers, offer promising pathways to improve fruit quality, yet field-to-greenhouse consistency and management-dependent responses still limit widely transferable soil-based recommendations.

Large quantities of crop residues are generated annually, and straw is a major fraction of these residues (Shinde et al., 2022). Crop straw decomposes slowly when directly incorporated into soil (Priya et al., 2025), and high C:N straw can temporarily reduce plant-available N due to microbial immobilization during early decomposition (van Rijssel et al., 2025). Fungal solid-state fermentation can partially degrade straw (Sufyan et al., 2022), yielding a fungal-fermented straw product (FSP) enriched with readily available nutrients and microbial metabolites; recent work shows that microbial fermentation can substantially transform corn straw composition and nutrient forms (Chen et al., 2024). When applied to soil, FSP can enhance organic matter content, nutrient availability, and microbial activity, potentially stimulating metabolic pathways for the synthesis of antioxidants (e.g., lycopene, ascorbic acid) and flavor compounds in tomato fruits. However, the effect of straw-derived inputs depends strongly on placement depth because depth controls residue–soil contact, oxygen and moisture regimes, and the vertical distribution of C and nutrients that shape microbial activity. Depth of residue incorporation has been shown to alter crop yield and the distribution of soil organic C and total N across soil layers (Liu et al., 2021), and straw incorporation associated with deeper tillage can shift microbial community characteristics throughout cultivated layers (Liu et al., 2022). However, excessive application may disrupt soil nutrient balance or promote the proliferation of antibiotic resistance genes (ARGs), posing ecological risks. Evidence from organic fertilizer inputs indicates that some organic amendments can increase the prevalence and transfer potential of typical ARGs in agricultural soils, highlighting the need to evaluate resistome-related risks when introducing straw-derived organic inputs (Wei et al., 2022).

Therefore, key knowledge gaps remain regarding how the FSP rate interacts with soil incorporation depth (here, 3 and 10 cm in a pot system) to balance fruit quality benefits against soil ecological risks. In this study, we tested whether FSP can improve tomato fruit nutritional quality through soil-mediated processes. We hypothesized that an appropriate FSP rate combined with deeper incorporation would (i) promote more favorable nutrient release and humification, (ii) support beneficial decomposer and plant-associated microbial taxa, and (iii) improve yield and fruit nutritional traits, while avoiding increases in ARG abundance compared with conventional fertilization.

## 2 Materials and methods

### 2.1 Preparation of fungal-fermented straw and soil characteristics

Corn straw was used as the base material to produce the fungal-fermented straw product (FSP). The straw was chopped into 2–3 cm pieces, and wood chips and soybean meal were added at a mass ratio of corn straw: wood chips: soybean meal = 20:7:1 (w/w) to adjust the carbon-to-nitrogen ratio to approximately 50:1 (Chen et al., 2021). The initial moisture content of the chopped straw was 65% ± 8% (fresh basis), and the substrate moisture was adjusted to 65% prior to inoculation. The mixture was autoclaved at 121°C for 2 h and then inoculated with *Auricularia cornea* cv. Yumuer (strain No. 2018091901), obtained from the Engineering Research Center of Edible and Medicinal Fungi, Ministry of Education, Jilin Agricultural University. Solid-state fermentation was conducted in polypropylene cultivation bags (approximately 1.4 kg wet substrate per bag) at 25°C in the dark, with ambient relative humidity maintained at 50–60%, for 30 days until the mycelium fully colonized the substrate. After fermentation, the substrate was dried at 50°C and ground into a fine powder.

The soil used for the pot experiment was collected from the Mushroom–Vegetable Rotation Base of Jilin Agricultural University (Jilin Province, China; 43.89°N, 125.32°E). The soil was air-dried, sieved through a 10-mesh (2 mm) screen, and homogenized before pot filling. No mineral fertilizer was added prior to the experiment to isolate the effects of FSP. The soil was not autoclaved to preserve the native microbial background. Basic physicochemical properties of the FSP and the cultivation soil are provided in the **Supplementary Materials** (**Supplementary Table S1**).

### 2.2 Tomato cultivation and FSP treatments

The experiment was conducted in a solar-heated greenhouse at Jilin Agricultural University (Jilin Province, China; 43.89°N, 125.32°E) from 20 April 2023 to 20 August 2023. During the trial, the greenhouse temperature ranged from 15 to 28°C, and the relative humidity ranged from 50 to 70%. Tomato plants were grown under natural sunlight. Tomatoes were grown in rectangular foam containers (57 cm × 42.5 cm × 30 cm; wall thickness 2.7 cm). Each container was filled with 20 kg of the prepared soil. The experiment followed a completely randomized design, with seven treatments and three replicate containers per treatment. Treatments were coded as WR (0% FSP), DS (0.5% at 10 cm), DM (2% at 10 cm), DH (5% at 10 cm), SS (0.5% at 3 cm), SM (2% at 3 cm), and SH (5% at 3 cm) (**Supplementary Table S2**).

To apply FSP, the calculated amount of FSP was mixed thoroughly with the designated soil layer before pot filling. This procedure ensured that FSP was distributed within the intended soil zone. Each replicate container contained four tomato seedlings planted evenly, with an approximate within-container spacing of ~20 cm between plants. Containers were arranged with ~30 cm spacing between adjacent containers to reduce mutual shading and facilitate uniform management. Drainage holes were made at the bottom of each container, and the holes were covered with nylon mesh to prevent soil loss while allowing free drainage.

Standard greenhouse management practices were followed for all treatments. Plants were irrigated uniformly to maintain the soil in a consistently moist condition without waterlogging, and pest control was conducted biologically without chemical pesticides to avoid adverse effects on soil microbiota.

### 2.3 Plant growth monitoring and harvest

Tomato plant growth was monitored throughout the season. Vegetative growth parameters were measured at the peak of fruiting, which occurred at approximately 100 days after transplanting. Stem diameter (at the 5th internode above the soil) was measured with digital calipers, while plant height (from the soil surface to the apical growing point) and canopy width (widest horizontal spread of foliage) were measured using a ruler or measuring tape. These measurements were taken from all four plants in each container and averaged per replicate.

Tomato fruits were harvested at full maturity for quality analysis. Full maturity was defined as the red-ripe stage, with the peel completely turned red. The total fruit yield per plant was recorded by weighing all ripe fruits from each plant, and values were averaged by treatment. A subset of representative fruits from each replicate (5–6 fruits) was immediately frozen in liquid nitrogen and stored at −80°C for subsequent nutrient and quality analyses to preserve labile compounds.

### 2.4 Fruit quality analysis

The flesh of the harvested tomatoes was subjected to homogenization and centrifugation for preparation. Soluble solid content (SSC), ascorbic acid (AsA), flavonoid content, and lycopene were measured. SSC was determined using a refractometer. AsA was quantified by the Fe^3+^ reduction colorimetric method, measuring absorbance at 534 nm (Du et al., 2012). Flavonoids were quantified using the alkaline sodium nitrite colorimetric method, and absorbance was measured at 510 nm using an M8500 UV–VISIBLE spectrophotometer (Zhishen et al., 1999). Lycopene was quantified using high-performance liquid chromatography (HPLC). A 1 g sample was extracted by ultrasound and concentrated by nitrogen blowing, using methanol: isopropanol (4:6, v/v) as the mobile phase at a flow rate of 0.8 mL/min, with detection at 472 nm based on peak area (Berra, 2012).

### 2.5 Soil sampling and physicochemical analysis

Soil samples were collected at two time points: before transplanting (baseline) and at final harvest. At final harvest, rhizosphere soil was collected from each replicate container. Plants were gently removed, and loosely attached soil was first removed by shaking. Rhizosphere soil was then obtained by brushing the soil that remained tightly adhered to the roots into sterile bags. For each treatment, one composite rhizosphere sample was prepared per replicate container by pooling rhizosphere soil from the four plants within that container, resulting in three composite samples per treatment (n = 3).

Each composite sample was divided into subsamples. One subsample was air-dried and sieved through a 100-mesh screen for physicochemical analyses. Soil organic matter (OM), total nitrogen (TN), available phosphorus (AP), available potassium (AK), and humic fractions were determined following (Kooch et al., 2023) (with minor modifications where necessary). Specifically, OM was determined by potassium dichromate–concentrated sulfuric acid oxidation titration, TN was determined by the Kjeldahl method, soluble organic nitrogen was analyzed by potassium persulfate oxidation combined with UV–visible spectrophotometry, AP was determined by the molybdenum–antimony colorimetric method, and AK was measured using a flame photometer. Humic substances (humic acid, fulvic acid, and humin/humin carbon) were extracted using a pyrophosphate–sodium hydroxide solution.

### 2.6 Soil enzyme activity assays

Rhizosphere soil samples were stored at −80°C and thawed before enzyme assays. Activities of urease (S-UE), dehydrogenase (S-DHA), and nitrite reductase (S-NiR) were determined using commercial microplate assay kits (Suzhou Michy Bio, Suzhou, China) and a 96-well microplate reader, following the manufacturer’s instructions. Urease (S-UE) activity was measured by the indophenol blue colorimetric method based on NH_3_–N produced from urea hydrolysis. Briefly, 0.05 g soil was incubated at 37°C for 24 h, followed by centrifugation, and the supernatant was color-developed and read at 578 nm (Guo et al., 2012). Dehydrogenase (S-DHA) activity was determined using 2,3,5-triphenyl tetrazolium chloride (TTC) as the electron acceptor, which is reduced to triphenyl formazan (TF), and absorbance was read at 485 nm. Soil (0.1 g) was incubated at 37°C for 24 h, and the colored product was extracted following the kit protocol. Nitrite reductase (S-NiR) activity was quantified based on the decrease in nitrite participating in the diazotization color reaction, with absorbance measured at 540 nm. In brief, 0.04 g soil was reacted at 25°C for 1 h, followed by centrifugation, color development in 96-well plates, and absorbance reading at 540 nm.

### 2.7 Soil microbial community and ARG analysis

To examine the shifts in soil microbial communities due to FSP, we performed high-throughput DNA sequencing of 16S rRNA genes (for bacteria) and ITS regions (for fungi) on final-harvest rhizosphere soil samples. Seven treatments, each with three biological replicates (21 samples in total), were analyzed. Total genomic DNA was extracted from 0.5 g of fresh rhizosphere soil using a CTAB-based protocol with bead-beating to facilitate cell lysis. DNA quality was assessed by agarose gel electrophoresis and spectrophotometry. The V3-V4 region of the bacterial 16S rRNA gene was PCR-amplified using primers 338F/806R, while the fungal ITS1 region was amplified with primers ITS1F/ITS2R. Amplicons from all samples were barcoded, pooled in equimolar amounts, and sequenced on an Illumina NovaSeq 6000 platform (PE250 mode).

Raw reads were processed in QIIME2 (Bolyen et al., 2019): demultiplexed, quality-filtered, and clustered into operational taxonomic units (OTUs) at 97% similarity (Rognes et al., 2016). Representative sequences were taxonomically assigned using the SILVA (bacteria) (Quast et al., 2013) and UNITE (fungi) databases (Nilsson et al., 2019). Microbial diversity indices (Shannon diversity, richness) and community composition were calculated for each treatment. Beta diversity was analyzed via principal coordinates analysis (PCoA) based on Bray-Curtis distances to visualize community differences.

In addition to community profiling, we evaluated the presence of antibiotic resistance genes (ARGs) in soil as an ecological safety consideration. A subset of DNA samples was subjected to high-throughput shotgun metagenomic sequencing (NovaSeq platform). Metagenomic reads were searched against a comprehensive ARG database (e.g., CARD) (Alcock et al., 2020) to identify genes conferring antibiotic resistance. Detected ARGs were categorized by resistance mechanism (e.g., efflux pump, target modification, antibiotic inactivation).

### 2.8 Statistical analysis

All experimental data were analyzed using statistical software (SPSS 26.0, SPSS Inc., USA). One-way analysis of variance (ANOVA) was performed to test for significant differences among the seven treatments. For each measured parameter, the treatment means were compared using Tukey’s honest significant difference (HSD) *post-hoc* test at a significance level of *p* < 0.05. Data are presented as mean ± standard error (SE) of three replicates (unless otherwise noted). Graphical visualizations were produced using OriginPro 2023 (OriginLab, USA) and annotated with letters to indicate statistical groupings. For microbial community data, multivariate statistical analysis (PERMANOVA) was applied to determine if community composition differed significantly by treatment (*p* < 0.05).

## 3 Results

### 3.1 Tomato growth and yield response to FSP

Treatments were coded as WR (0% FSP), DS (0.5% at 10 cm), DM (2% at 10 cm), DH (5% at 10 cm), SS (0.5% at 3 cm), SM (2% at 3 cm), and SH (5% at 3 cm). Tomato growth and yield responses are summarized in **Table 1**. Plant height ranged from 24.35 in WR to 26.93 in SM, and SM was significantly higher than WR; the other treatments were not significantly different from WR. Stem diameter differed among treatments. WR (7.10) and DH (6.73) were not significantly different, while SS showed the lowest stem diameter at 4.95 and was significantly lower than WR. Canopy width also varied across treatments: WR (26.72) and DH (26.03) were not significantly different, whereas DM (23.11) and SH (23.01) were significantly lower than WR, and SS had the smallest canopy width at 20.51, significantly lower than WR.

**Table 1:** Effects of fungal-fermented straw product (FSP) treatments on tomato growth and yield.

FSP treatments	Stem diameter (mm)	Plant height (cm)	Canopy width (cm)	Yield (g per plant)
WR	7.10 ± 0.78 a	24.35 ± 2.25 b	26.72 ± 3.45 a	309.50 ± 21.67 b
DH	6.73 ± 0.76 a	24.73 ± 1.67 ab	26.03 ± 2.79 ab	398.40 ± 13.94 a
DM	6.33 ± 0.94 ab	26.12 ± 1.86 ab	23.11 ± 2.67 cd	400.68 ± 11.37 a
DS	5.87 ± 0.41 b	24.93 ± 1.76 ab	23.56 ± 3.09 bcd	315.27 ± 11.47 b
SH	5.74 ± 1.15 bc	25.09 ± 2.81 ab	23.01 ± 3.82 cd	389.70 ± 15.82 a
SM	5.64 ± 1.28 bc	26.93 ± 1.65 a	24.37 ± 2.71 abc	314.84 ± 15.33 b
SS	4.95 ± 0.77 c	24.87 ± 1.25 ab	20.51 ± 2.31 d	311.31 ± 9.52 b

Treatments were coded as WR (0% FSP), DS (0.5% at 10 cm), DM (2% at 10 cm), DH (5% at 10 cm), SS (0.5% at 3 cm), SM (2% at 3 cm), and SH (5% at 3 cm). Data are expressed as mean ± standard error (n=3), and different lowercase letters indicate significant differences among treatments (*p* < 0.05).

Yield per plant differed clearly among treatments (**Table 1**). The highest yields occurred in DM (400.68), DH (398.40), and SH (389.70); these three treatments were significantly higher than WR (309.50). In contrast, DS (315.27), SM (314.84), and SS (311.31) did not differ significantly from WR. Dose–response analysis showed significant positive relationships between FSP rate and yield under both incorporation depths (**Supplementary Figure S1a**). The regression was stronger under shallow incorporation than deep incorporation.

During the greenhouse trial, no visible symptoms of phytotoxicity, salt injury, or abnormal growth were observed in any treatment.

### 3.2 Improvement of tomato fruit nutritional quality

**Figure 1:**
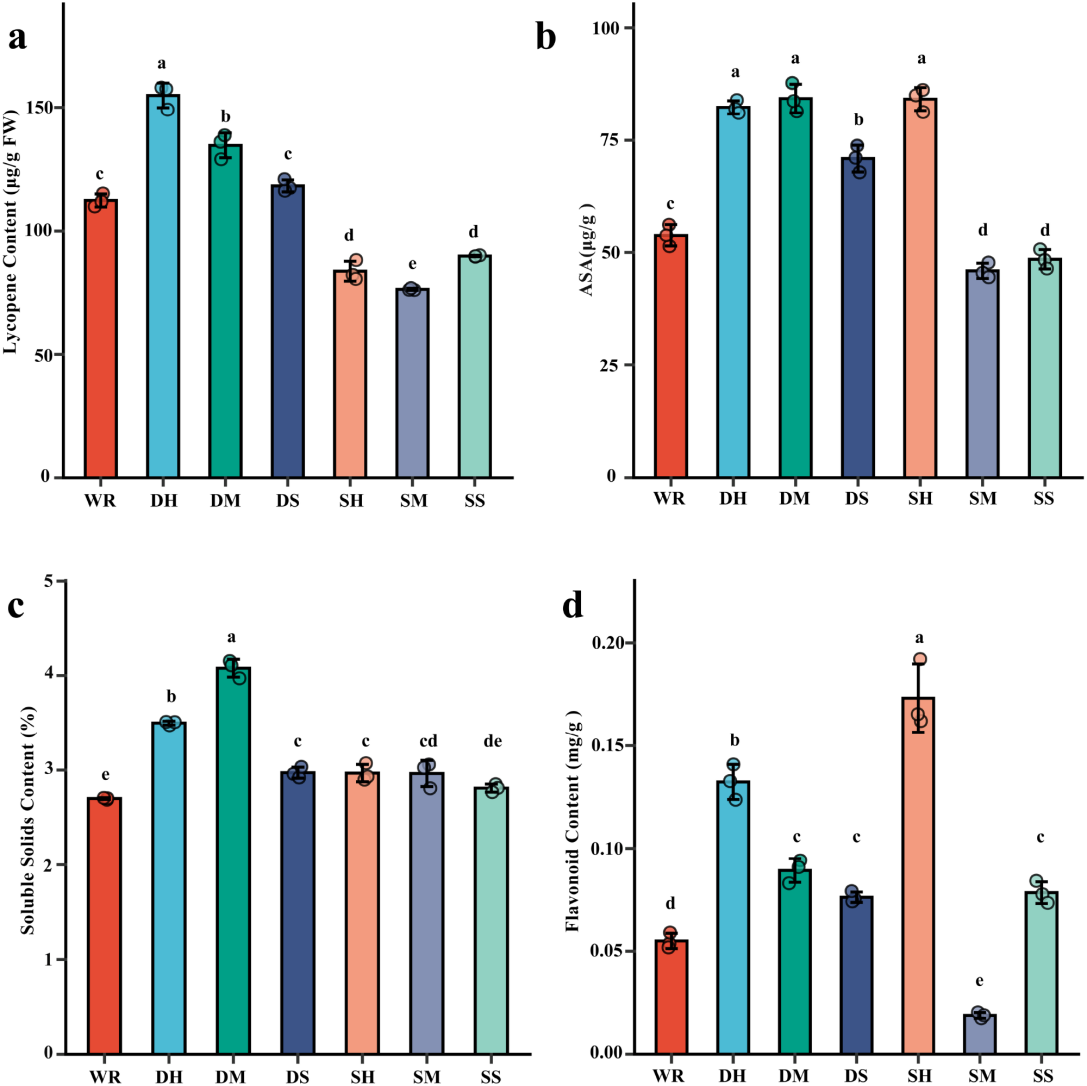
Effect of different FSP treatments on tomato fruit quality parameters. **(a)** Lycopene content (μg/g FW), **(b)** Ascorbic acid content (μg/g), **(c)** Soluble solids content (%), **(d)** Flavonoid content (mg/g). WR, 0% FSP, DS, 0.5% FSP at 10 cm soil depth, DM, 2% FSP at 10 cm soil depth, DH, 5% FSP at 10 cm soil depth, SS, 0.5% FSP at 3 cm soil depth, SM, 2% FSP at 3 cm soil depth, SH, 5% FSP at 3 cm soil depth. Data are mean ± SE (n = 3). Different lowercase letters above bars indicate significant differences among treatments (Tukey’s HSD, *p* < 0.05).

Key fruit nutritional traits are presented in **Figure 1**. Lycopene differed significantly among treatments. DH reached 154.86 and was significantly higher than all other treatments. DM reached 134.57 and was significantly higher than WR (112.31), while DS (118.18) did not differ significantly from WR. SH (83.55) and SS (89.68) were significantly lower than WR, and SM (76.26) was the lowest and significantly lower than WR.

Ascorbic acid (AsA) also varied strongly. DH (82.36), DM (84.32), and SH (84.18) shared the highest group and were all significantly higher than WR (53.89). DS reached 70.93 and was significantly higher than WR but significantly lower than the DH/DM/SH group. SM (45.94) and SS (48.51) were significantly lower than WR.

Soluble solids content (SSC) showed a clear treatment effect. DM reached 4.08 and was significantly higher than all other treatments. DH reached 3.50 and was significantly higher than WR (2.70). DS (2.97) and SH (2.97) did not differ significantly from each other, and SM (2.96) was not significantly different from DS/SH. SS reached 2.81 and did not differ significantly from WR, but was significantly lower than DS and SH. Dose–response analysis indicated a significant positive relationship between FSP rate and SSC under shallow incorporation, while the relationship was weaker under deep incorporation (**Supplementary Figure S1b**).

Flavonoids were highest in SH (0.173), which was significantly higher than all other treatments. DH reached 0.132 and was significantly higher than DM (0.089), while DM did not differ significantly from DS (0.076) or SS (0.078); all three (DM/DS/SS) were significantly higher than WR (0.055). SM was the lowest at 0.019 and was significantly lower than WR.

### 3.3 Changes in soil nutrient status and enzyme activities with FSP

**Figure 2:**
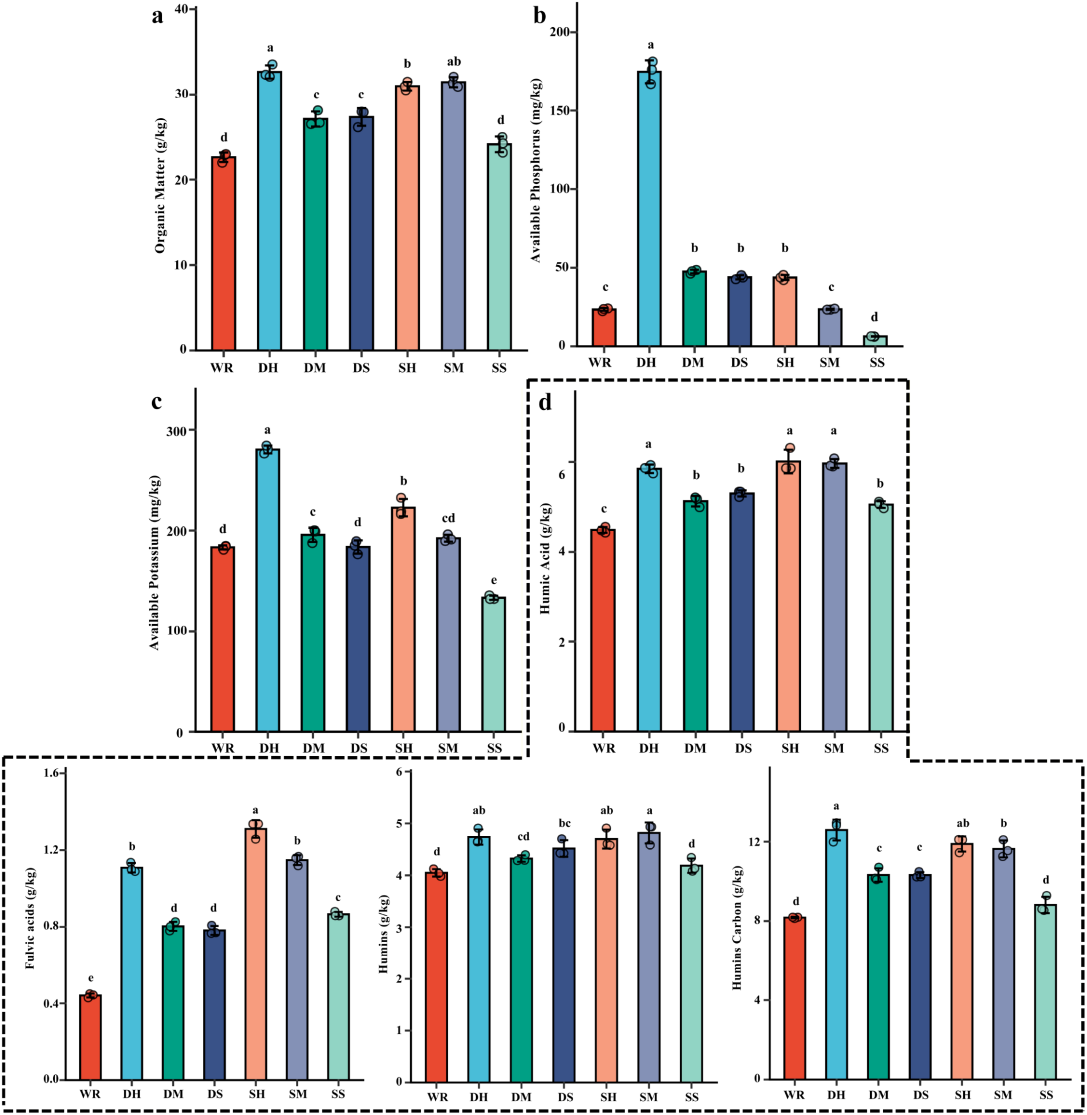
Effects of different FSP treatments on soil organic matter and nutrient fractions at tomato harvest. **(a)** Organic matter content (g/kg), **(b)** Available phosphorus (mg/kg), **(c)** Available potassium (mg/kg), **(d)** Humic fractions, including humic acid (g/kg), fulvic acid (g/kg), humins (g/kg), humins carbon (g/kg). WR, 0% FSP, DS, 0.5% FSP at 10 cm soil depth, DM, 2% FSP at 10 cm soil depth, DH, 5% FSP at 10 cm soil depth, SS, 0.5% FSP at 3 cm soil depth, SM, 2% FSP at 3 cm soil depth, SH, 5% FSP at 3 cm soil depth. Data are mean ± SE (n = 3). Different lowercase letters above bars indicate significant differences among treatments (Tukey’s HSD, *p* < 0.05).

Soil nitrogen forms are summarized in **Table 2**, and other soil indicators are presented in **Figure 2**. Total nitrogen differed among treatments. DH and SM reached 1.38 and were significantly higher than WR (1.08), while SS (1.07) did not differ significantly from WR; DM (1.14) and DS (1.16) were intermediate.

**Table 2:** Effects of fungal-fermented straw product (FSP) treatments on soil nitrogen content.

FSP treatments	Total nitrogen (g/kg)	Ammonium nitrogen (mg/kg)	Nitrate nitrogen (mg/kg)	DON (mg/kg)
WR	1.08 ± 0.02 cd	2.56 ± 0.13 c	4.21 ± 0.29 e	16.74 ± 1.43 a
DH	1.38 ± 0.04 a	3.67 ± 0.23 a	13.83 ± 0.17 c	14.96 ± 0.88 b
DM	1.14 ± 0.08 bc	3.20 ± 0.09 a	9.54 ± 0.51 d	11.94 ± 0.87 cd
DS	1.16 ± 0.07 b	3.10 ± 0.32 abc	2.96 ± 0.11 f	10.32 ± 0.30 e
SH	1.33 ± 0.03 a	3.18 ± 0.10 ab	17.98 ± 0.17 a	12.60 ± 0.37 c
SM	1.38 ± 0.02 a	2.62 ± 0.09 bcd	15.58 ± 0.31 b	10.66 ± 0.69 de
SS	1.07 ± 0.01 cd	2.24 ± 0.09 d	2.78 ± 0.12 f	10.07 ± 0.79 e

Treatments were coded as WR (0% FSP), DS (0.5% at 10 cm), DM (2% at 10 cm), DH (5% at 10 cm), SS (0.5% at 3 cm), SM (2% at 3 cm), and SH (5% at 3 cm). Soil nitrogen content, including TN, NH_4_^+^, NO_3_^−^, and DON. Data are expressed as mean ± standard error (n=3), and different lowercase letters indicate significant differences among treatments (*p* < 0.05).

Ammonium nitrogen also varied. DH (3.67) and DM (3.20) were in the highest group, while WR (2.56) was lower; SS (2.24) was significantly lower than WR. Nitrate nitrogen showed the strongest separation: SH reached 17.98 and was significantly higher than all other treatments, followed by SM at 15.58 and DH at 13.83; WR was 4.21. DS (2.96) and SS (2.78) were the lowest group. Dissolved organic nitrogen decreased in all FSP treatments relative to WR (16.74), with DS and SS in the lowest group.

Soil organic matter increased in most treatments (**Figure 2a**). DH (32.65) was in the highest group and was significantly higherxthan WR (22.62). SH (30.98) and SM (31.45) were also significantly higher than WR. DM (27.10) and DS (27.34) were significantly higher than WR, while SS (24.14) did not differ significantly from WR.

Soil available phosphorus changed markedly (**Figure 2b**). DH reached 174.83 and was significantly higher than all other treatments. DM (47.56), DS (43.91), and SH (43.77) formed the next group and were significantly higher than WR (23.43). SM (23.56) did not differ significantly from WR, while SS (6.30) was significantly lower than WR.

Soil available potassium also differed (**Figure 2c**). DH reached 280.33 and was significantly higher than all other treatments. SH reached 222.67 and was significantly higher than WR (183.33). DM reached 195.67 and was significantly higher than WR, whereas DS (183.67) did not differ significantly from WR; SM (192.33) was intermediate. SS (133.33) was significantly lower than WR.

Humic fractions increased in several treatments (**Figure 2d**). For humic acid, DH, SH, and SM were in the highest group, while WR was lower. Fulvic acid increased most in SH, and WR was the lowest group. Humin and humin carbon were highest in deep/high-related treatments (notably DH), while WR and SS were in the lower group.

**Figure 3:**
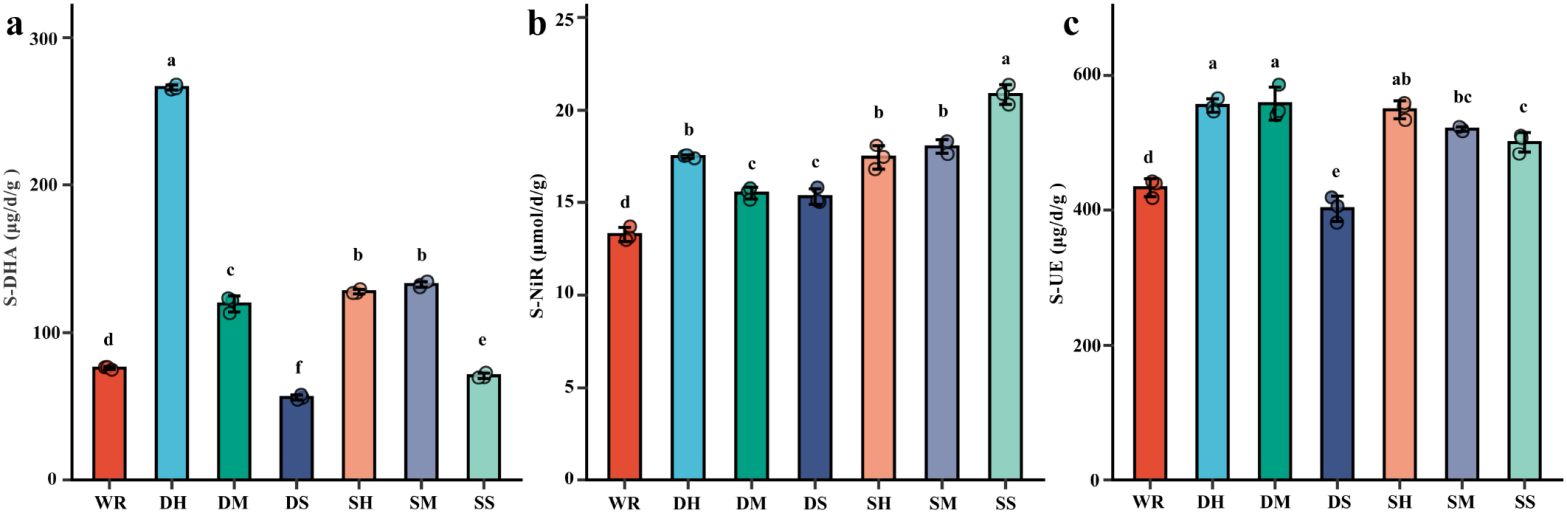
Effect of different FSP treatments on soil enzyme activities. **(a)** S-DHA, **(b)** S-NIR, **(c)** S-UE. WR, 0% FSP, DS, 0.5% FSP at 10 cm soil depth, DM, 2% FSP at 10 cm soil depth, DH, 5% FSP at 10 cm soil depth, SS, 0.5% FSP at 3 cm soil depth, SM, 2% FSP at 3 cm soil depth, SH, 5% FSP at 3 cm soil depth. Data are mean ± SE (n = 3). Different lowercase letters above bars indicate significant differences among treatments (Tukey’s HSD, *p* < 0.05).

Soil enzyme activities are presented in **Figure 3**. Dehydrogenase (S-DHA) was highest in DH at 266.27 and was significantly higher than all other treatments. SH (127.76) and SM (132.57) were significantly higher than DM (119.46), and WR (76.12) was significantly higher than SS (70.88) and DS (56.27); DS was the lowest group.

Nitrite reductase (S-NiR) increased from 13.27 in WR to 20.85 in SS, and SS was significantly higher than all other treatments. DH (17.47), SH (17.43), and SM (18.03) were significantly higher than WR, while DM (15.49) and DS (15.31) were intermediate.

Urease (S-UE) differed among treatments. DH (555.50) and DM (558.28) were in the highest group and were significantly higher than WR (432.94). SH (549.22) was not significantly different from DH/DM, while SM (520.67) and SS (500.67) were lower than DH/DM but higher than WR. DS (401.89) was the lowest group.

### 3.4 Soil microbial community shifts and ARG profiles

PCoA based on 16S/ITS amplicon profiles separated the FSP-treated soils from the control WR (**Supplementary Figure S2a**). WR samples clustered apart from all FSP treatments. The deep-incorporation treatments (DM and DH) clustered more tightly than the shallow-incorporation treatments. Among shallow treatments, SH showed greater dispersion in ordination space (**Supplementary Figure S2a**).

**Figure 4:**
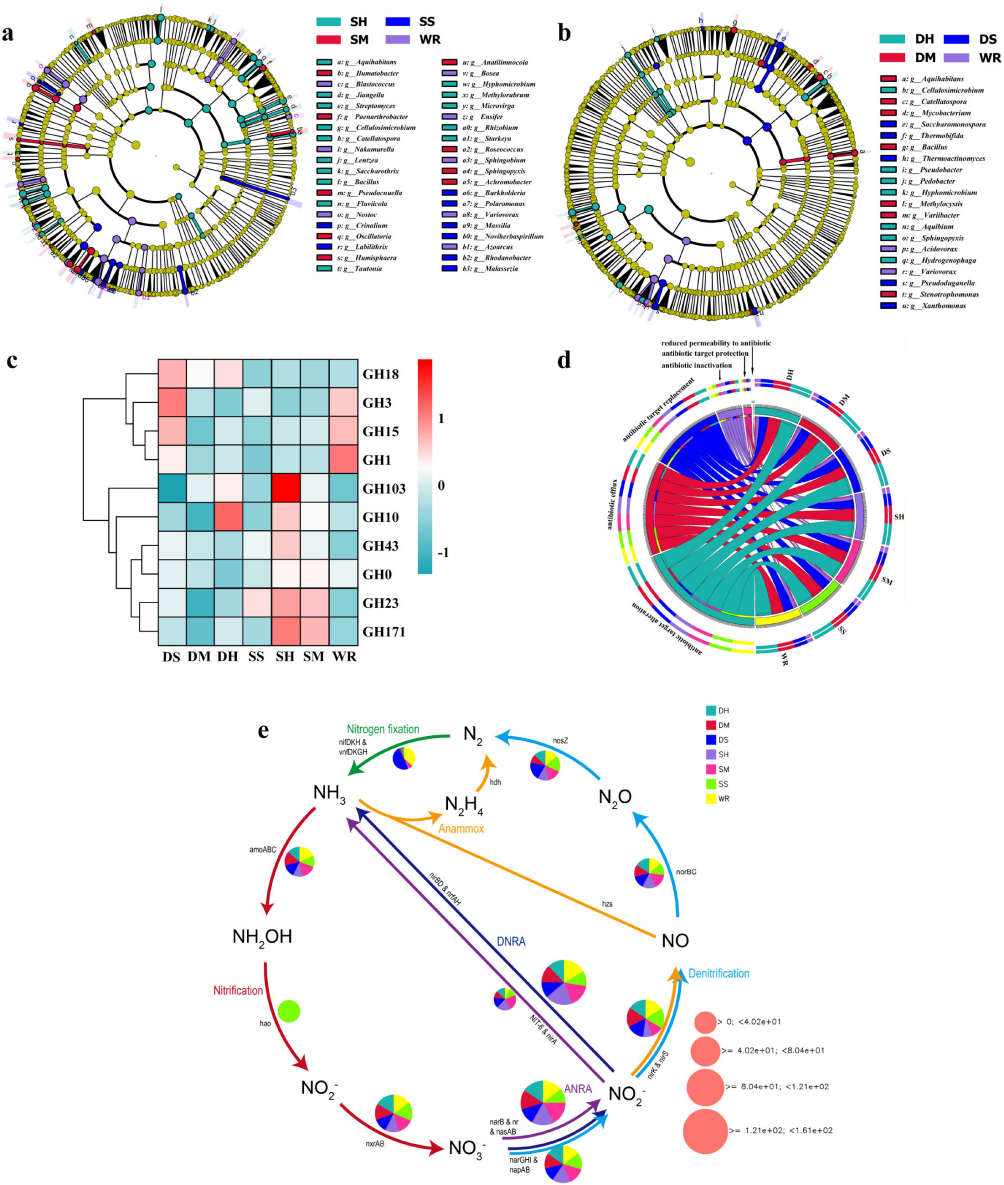
Effects of fungal-fermented straw product (FSP) treatments on soil microbial community composition, functional genes, and nitrogen cycle pathways. **(a)** Circular cladogram of bacterial taxa showing differences among shallow-incorporated treatments and control, **(b)** Circular cladogram of bacterial taxa for deep-incorporated treatments and control, **(c)** Heatmap of glycoside hydrolase (GH) family gene abundance across treatments, **(d)** Circos plot showing associations between antibiotic resistance gene (ARG) categories and bacterial taxa, **(e)** Schematic diagram of nitrogen transformation pathways (nitrification, denitrification, anammox, DNRA) with pie charts indicating the relative abundance of functional genes in each treatment, bubble size reflects the total abundance of related genes. WR, 0% FSP, DS, 0.5% FSP at 10 cm soil depth, DM, 2% FSP at 10 cm soil depth, DH, 5% FSP at 10 cm soil depth, SS, 0.5% FSP at 3 cm soil depth, SM, 2% FSP at 3 cm soil depth, SH, 5% FSP at 3 cm soil depth.

At the genus level, WR was characterized by higher relative representation of oligotrophic taxa such as *Bradyrhizobium* and *Variovorax* (**Supplementary Figure S2b**). In contrast, FSP treatments showed higher relative abundances of *Streptomyces*, *Sphingomonas*, *Nocardioides*, and *Arthrobacter*, with the enrichment more evident under deep placement (**Figures 4a, b**, **Supplementary Figure S2b**).

Redundancy analysis linked community variation to soil functional indicators (**Supplementary Figure S2c**). RDA1 and RDA2 explained 49.53% and 17.06% of the variance, respectively. The ordination patterns aligned with dehydrogenase (DHA) and urease (UE) activities and with humic fractions (Hs, HAs, FAs, HC). Correlation analysis further supported these relationships (**Supplementary Figure S2d**): *Streptomyces*, *Nocardioides*, *Sphingomonas*, and *Arthrobacter* showed significant positive correlations with DHA, UE, and humic components, whereas *Bradyrhizobium* and *Variovorax* showed weak or negative associations.

Shotgun metagenomic profiling indicated shifts in functional genes after FSP application (**Figure 4c**). Genes encoding glycoside hydrolase (GH) families were more abundant in FSP treatments, particularly in the moderate and high deep applications. Several GH families related to plant cell-wall depolymerization (e.g., GH10, GH23, GH103, GH171) showed higher relative abundance under deep placement (**Supplementary Figure S3a**). Correlation analysis indicated that GH103, GH10, and GH171 were positively associated with DHA, UE, and humic fractions (Hs, HAs, FAs, HC), while GH3 and GH15 showed weaker or negative associations (**Supplementary Figure S3b**).

ARG profiles differed among treatments (**Figure 4d**). ARGs were classified by resistance mechanisms, including efflux pump, target modification, and antibiotic inactivation. WR showed a relatively higher proportion of antibiotic inactivation–related genes, whereas high-dose FSP treatments increased the relative share of efflux-type ARGs. The highest efflux-type proportions occurred in SH and DH, reaching approximately 38% of detected ARGs, compared with approximately 30% in WR. SH also showed an increase in total ARG abundance, while DM exhibited only minor changes relative to WR. Consistent with this pattern, the mechanism–environment heatmap (**Supplementary Figure S3c**) shows positive associations between the efflux category and labile carbon transformation (higher FAs, DHA) and with humus accumulation (HC/HAs), while antibiotic inactivation tends to correlate negatively with humic fractions.

Pathway-level mapping of nitrogen-cycling genes indicated treatment-associated differences (**Figure 4e**). Deep placement (DM/DH) showed higher relative representation of denitrification markers (nirK/nirS → norB/C → nosZ) and nitrogen-fixation genes, whereas SS showed relatively stronger signals for nitrification-related genes (amo/hao).

## 4 Discussion

FSP application generated a clear yield and quality signal under greenhouse conditions, but the response depended on both rate and incorporation depth. Yield increased strongly under DM, and DH/SH also produced higher yields than WR, while DS/SM/SS showed little change. The dose–response patterns (**Supplementary Figure S1**) support a generally positive association between FSP rate and yield under both depths, yet the trait-by-trait responses indicate that “more” is not always “better” for overall performance. From a practical standpoint, DM provided the most balanced outcome across yield and multiple fruit quality traits, but this should be treated as a cautious recommendation because greenhouse systems reduce leaching, buffer temperature and moisture fluctuations, and simplify spatial heterogeneity relative to field soils.

The fruit quality responses help identify likely drivers. Lycopene increased most clearly under deep incorporation at higher rates, consistent with the importance of plant nutritional status—especially phosphorus and energy metabolism—in carotenoid accumulation (Wang et al., 2021). AsA increased strongly in DM/DH/SH, which is consistent with improved nutrient supply and redox buffering, and parallels work showing that antioxidant-related traits in tomato can respond to amendments that improve stress tolerance and nutrient availability (Ikram et al., 2024). SSC peaked under DM, consistent with the central role of potassium in sugar transport and fruit filling (Liu et al., 2021). The flavonoid pattern, with the highest level under SH, fits the broader view that phenolics can rise when plants experience mild trade-offs or localized rhizosphere constraints, even when yield is not necessarily reduced (Cosme et al., 2025). These results also align with reports that organic amendments such as vermicompost products can improve tomato fruit quality traits and antioxidant capacity in greenhouse systems (Ávila-Juárez et al., 2015; Lara-Capistrán et al., 2024). Importantly, dataset shows that different quality traits peak under different FSP regimes (e.g., DH for lycopene, DM for SSC), so the “best” treatment depends on the target outcome and the acceptable level of ecological perturbation.

**Figure 5:**
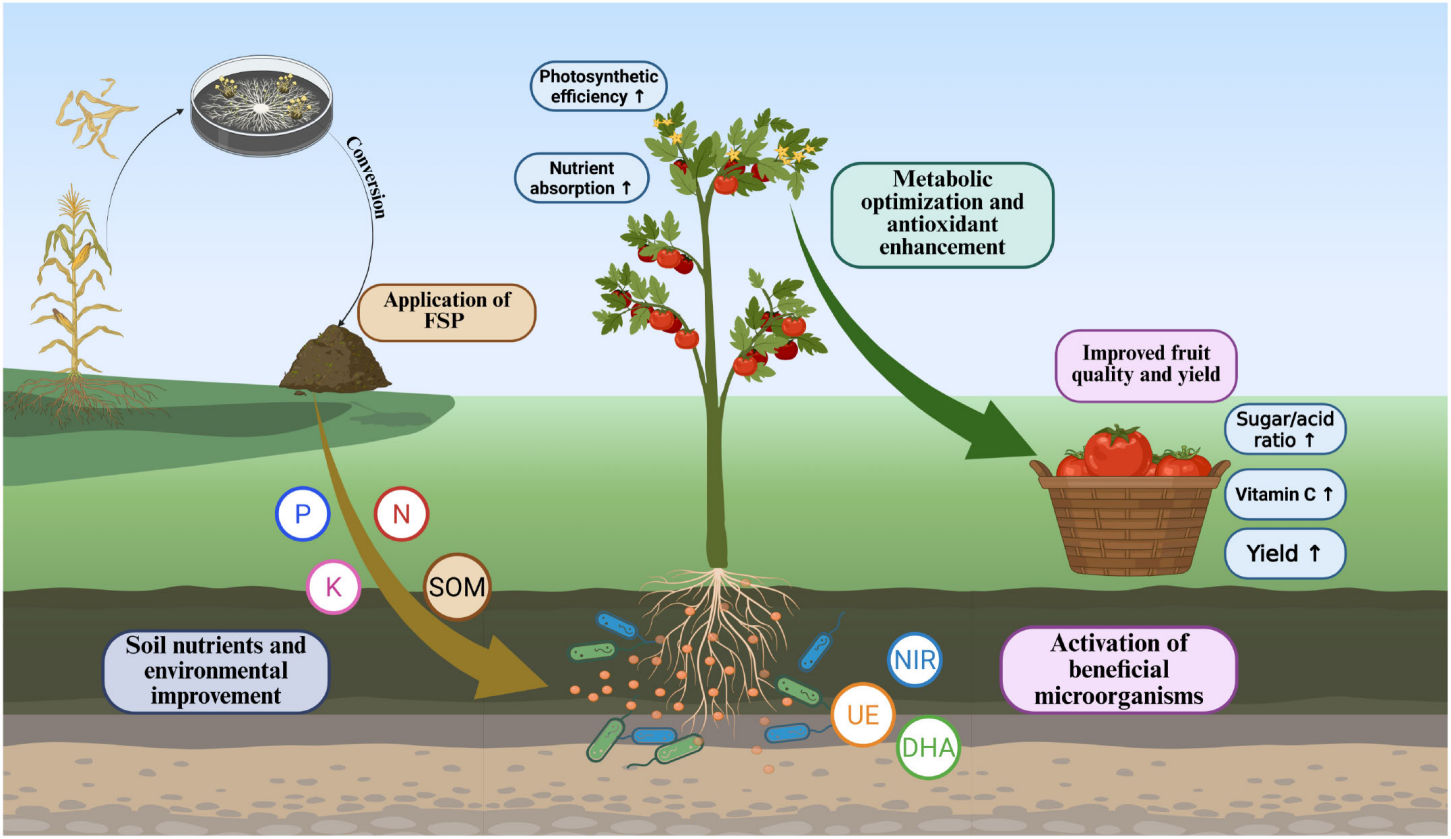
Proposed mechanism by which FSP application improves tomato fruit quality and yield. FSP is produced by fungal fermentation of crop straw and applied to soil, enhancing soil nutrients (organic matter, N, P, K) and improving the soil environment. This promotes nutrient absorption and photosynthetic efficiency, activates beneficial microorganisms, and increases soil enzyme activities. These changes optimize plant metabolism and enhance antioxidant production, resulting in higher sugar/acid ratio, increased vitamin C, and improved yield in tomato fruits.

Soil nutrient shifts and enzyme activities provide mechanistic support for the plant responses. Increases in soil organic matter and humic-related fractions are consistent with improved soil buffering and nutrient supply capacity that can support productivity (Xing et al., 2025). Enzyme activities (DHA, NiR, UE) reflect microbial functional changes linked to nutrient cycling and organic matter turnover; in soil systems, these enzyme indicators are widely used to interpret microbial processes under changing conditions (Burns et al., 2013). Higher DHA and UE under DM/DH suggest intensified microbial respiration and N mineralization potential, which can help sustain plant-available nutrients during fruit development (Pan et al., 2023). Depth-dependent N patterns also matter: shallow high rate produced very high nitrate, while deep placement generally showed a more moderated profile. Such contrasts can become more important in fields where rainfall-driven transport and heterogeneous aeration can amplify nitrate loss pathways. The broader microbial shifts toward decomposer/PGP-associated taxa (e.g., *Streptomyces*, *Sphingomonas*, *Nocardioides*, *Arthrobacter*) provide an additional biological link to decomposition and nutrient release; Streptomyces in particular has been repeatedly reported as a plant-growth-promoting group with measurable impacts on crop traits (Srinivas et al., 2022). Together with the increased GH-family potential reported in results, these patterns support the integrated mechanism summarized in **Figure 5**: FSP inputs improve nutrient pools and soil organic matter status, stimulate microbial decomposition and enzyme-mediated cycling, and thereby support higher yield and fruit nutritional quality through improved plant nutrition and allocation. Although no visible phytotoxicity or salt injury symptoms were observed under greenhouse conditions, field validation is still needed because open-field environments may amplify salinity-related or other stress responses.

Ecological safety signals need to be weighed alongside agronomic gains. ARG profiles shifted most under high-rate treatments, with an increased relative share of efflux-type ARGs, while DM showed only minor changes relative to WR. This pattern is consistent with the general understanding that composting and amendment use can show complex ARG dynamics, including persistence, attenuation, or rebound depending on substrates, microbial succession, and environmental conditions (Ma et al., 2025; Ukachi et al., 2025). Because ARG-related outcomes can have downstream implications, it is also reasonable to frame results in terms of potential exposure pathways and risk context for compost-derived products. These considerations reinforce why DM should be presented as a cautious recommendation rather than a universal prescription: it delivered strong agronomic outcomes with comparatively smaller ARG shifts in this greenhouse study, but field verification remains necessary. In practice, field trials should explicitly test whether DM maintains yield–quality benefits under rainfall and open-field heterogeneity, while tracking nitrate dynamics and ARG trajectories across time. Finally, it is useful to position FSP within the wider literature on mushroom-related organic inputs. Spent mushroom compost and related substrates have been studied (Zhang et al., 2025), and as greenhouse amendments for tomato, often improving growth and/or fruit quality (Çelikel, 1999), and straw–substrate composting strategies have been optimized to promote effective decomposition and reuse (Ma et al., 2023). In this context, FSP represents a biologically active, pre-decomposed straw-based input that can produce rapid, measurable improvements after one season in a controlled environment, but the best-supported claim at this stage is conditional: DM appears most promising among the tested treatments, and the next step is to confirm performance and safety outcomes under field conditions.

## 5 Conclusion and practical implications

Applying fungal-fermented straw at about 2% of soil weight and burying it 10 cm deep proved optimal. Under this practice tomato fruits contained roughly 40% more lycopene, 20-40% more vitamin C and soluble sugars, and higher flavonoids, while yield rose by about 30% compared with untreated soil. At the same time, soil organic matter, available phosphorus and potassium, and enzyme activities all increased, and beneficial microbes became more abundant. Ecological safety was acceptable: antibiotic-resistance gene markers rose only when a heavy 5% dose was left near the surface, not under the recommended moderate, deep placement. Thus, fungal-fermented straw turns crop residue into a useful amendment that raises both fruit quality and soil fertility without added risk when applied at the right rate and depth. Wider adoption could give growers higher-value produce and healthier soils, while supporting sustainable agriculture goals. We hypothesized that the agronomic benefits and ecological safety of FSP depend jointly on application rate and incorporation depth, via coordinated changes in soil nutrient pools, enzyme-mediated cycling, and the rhizosphere microbiome; the present results support this rate × depth framework under greenhouse conditions. Future studies should validate DM under open-field conditions across soil types and seasons, quantify salinity-related indicators (e.g., EC) and nitrate loss pathways, link depth effects to root traits/root activity, and track longer-term SOM stabilization and ARG trajectories.

## Data Availability

The original data presented in the study are deposited in the NCBI repository under accession number PRJNA1422982: https://www.ncbi.nlm.nih.gov/bioproject/PRJNA1422982.

## References

[B1] Alcock B. P. Raphenya A. R. Lau T. T. Y. Tsang K. K. Bouchard M. Edalatmand A. . (2020). CARD 2020: antibiotic resistome surveillance with the comprehensive antibiotic resistance database. Nucleic Acids Res. 48, D517–D525. doi: 10.1093/nar/gkz935, PMID: 31665441 PMC7145624

[B2] Ávila-Juárez L. Rodríguez González A. Rodríguez Piña N. Guevara González R. G. Torres Pacheco I. Ocampo Velázquez R. V. . (2015). Vermicompost leachate as a supplement to increase tomato fruit quality. J. Soil Sci. Plant Nutr. 15, 46–59. doi: 10.4067/S0718-95162015005000005, PMID: 27315006

[B3] Berra W. G. (2012). HPLC method optimization and validation for determination of lycopene in tomato (lycopersicon esculentum, mill.) fruits. Sci. Technol. Arts Res. J. 1, 14–26. doi: 10.4314/star.v1i4.98810

[B4] Bolyen E. Rideout J. R. Dillon M. R. Bokulich N. A. Abnet C. C. Al-Ghalith G. A. . (2019). Reproducible, interactive, scalable and extensible microbiome data science using QIIME 2. Nat. Biotechnol. 37, 852–857. doi: 10.1038/s41587-019-0209-9, PMID: 31341288 PMC7015180

[B5] Burns R. G. DeForest J. L. Marxsen J. Sinsabaugh R. L. Stromberger M. E. Wallenstein M. D. . (2013). Soil enzymes in a changing environment: current knowledge and future directions. Soil Biol. Biochem. 58, 216–234. doi: 10.1016/j.soilbio.2012.11.009, PMID: 41735180

[B6] Çelikel G. (1999). Effect of different substrates on yield and quality of tomato. Acta Hortic. 491, 353–356. doi: 10.17660/ActaHortic.1999.491.54

[B7] Chen J. Cai Y. Wang Z. Xu Z. Zhuang W. Liu D. . (2024). Solid-state fermentation of corn straw using synthetic microbiome to produce fermented feed: The feed quality and conversion mechanism. Sci. Total Environ. 920, 171034. doi: 10.1016/j.scitotenv.2024.171034, PMID: 38369147

[B8] Chen Y. Sossah F. L. Lv Z. Lv Y. Tian L. (2021). Effect of wheat bran and maize straw substrates on the agronomic traits and nutritional content of auricularia cornea cv. Yu muer. Sci. Hortic. 286, 110200. doi: 10.1016/j.scienta.2021.110200, PMID: 41735180

[B9] Cosme F. Aires A. Pinto T. Oliveira I. Vilela A. Gonçalves B. (2025). A comprehensive review of bioactive tannins in foods and beverages: functional properties, health benefits, and sensory qualities. Molecules 30, 800. doi: 10.3390/molecules30040800, PMID: 40005115 PMC11858154

[B10] Du J. Cullen J. J. Buettner G. R. (2012). Ascorbic acid: chemistry, biology and the treatment of cancer. Biochim. Biophys. Acta (bba)-rev Cancer 1826, 443–457. doi: 10.1016/j.bbcan.2012.06.003, PMID: 22728050 PMC3608474

[B11] Guo H. Yao J. Cai M. Qian Y. Guo Y. Richnow H. H. . (2012). Effects of petroleum contamination on soil microbial numbers, metabolic activity and urease activity. Chemosphere 87, 1273–1280. doi: 10.1016/j.chemosphere.2012.01.034, PMID: 22336736

[B12] Haraira A. a. Mazhar H. S.-u. Khalid M. N. Tariq M. Nazir S. Habib I. (2022). Enhancing health benefits of tomato by increasing its antioxidant contents through different techniques: a review. Adv. Life Sci. 9, 131–142. doi: 10.62940/als.v9i2.1352

[B13] Ikram M. Minhas A. AL-Huqail A. A. Ghoneim A. M. Mahmood S. Mahmoud E. . (2024). Promoting tomato resilience: effects of ascorbic acid and sulfur-treated biochar in saline and non-saline cultivation environments. BMC Plant Biol. 24, 1053. doi: 10.1186/s12870-024-05734-w, PMID: 39511477 PMC11545619

[B14] Kooch Y. Kartalaei Z. M. Haghverdi K. Praeg N. (2023). Soil function indicators are influenced by land use of different ages: a case study in a semi-arid region. Sci. Total Environ. 861, 160570. doi: 10.1016/j.scitotenv.2022.160570, PMID: 36462654

[B15] Lara-Capistrán L. Reyes A. G. Murillo-Amador B. Aquino-Bolaño E. N. Hernández-Adame L. Hernández-Montiel L. G. (2024). Effect of a marine bacterium and vermicompost on antioxidant properties and fruit quality of solanum lycopersicum L. Rev. Terra Latinoam. 42. doi: 10.28940/terra.v42i0.1845

[B16] Liu J. Hu T. Feng P. Yao D. Gao F. Hong X. (2021). Effect of potassium fertilization during fruit development on tomato quality, potassium uptake, water and potassium use efficiency under deficit irrigation regime. Agric. Water Manage. 250, 106831. doi: 10.1016/j.agwat.2021.106831, PMID: 41735180

[B17] Liu N. Li Y. Cong P. Wang J. Guo W. Pang H. . (2021). Depth of straw incorporation significantly alters crop yield, soil organic carbon and total nitrogen in the north China plain. Soil Tillage Res. 205, 104772. doi: 10.1016/j.still.2020.104772, PMID: 41735180

[B18] Liu X. Peng C. Zhang W. Li S. An T. Xu Y. . (2022). Subsoiling tillage with straw incorporation improves soil microbial community characteristics in the whole cultivated layers: a one-year study. Soil Tillage Res. 215, 105188. doi: 10.1016/j.still.2021.105188, PMID: 41735180

[B19] Ma Y. Liu Z. Xu S. Hong Y. Huang X. (2025). Cu+-enhanced composting for effective removal of ARGs: a mechanism-guided and environmentally friendly approach. Chem. Eng. J. 524, 169402. doi: 10.1016/j.cej.2025.169402, PMID: 41735180

[B20] Ma Y. Liu L. Zhou X. Tian T. Xu S. Li D. . (2023). Optimizing straw-rotting cultivation for sustainable edible mushroom production: composting spent mushroom substrate with straw additions. J. Fungi 9, 925. doi: 10.3390/jof9090925, PMID: 37755033 PMC10532571

[B21] Montgomery D. R. Biklé A. (2021). Soil health and nutrient density: beyond organic vs. conventional farming. Front. Sustain. Food Syst. 5. doi: 10.3389/fsufs.2021.699147, PMID: 41728591

[B22] Nilsson R. H. Larsson K.-H. Taylor A. F. S. Bengtsson-Palme J. Jeppesen T. S. Schigel D. . (2019). The UNITE database for molecular identification of fungi: handling dark taxa and parallel taxonomic classifications. Nucleic Acids Res. 47, D259–D264. doi: 10.1093/nar/gky1022, PMID: 30371820 PMC6324048

[B23] Pan Y. Yu S.-S. Xiao Z.-C. Min Y. Tian T. Zheng Y.-M. . (2023). Re-evaluation and modification of dehydrogenase activity tests in assessing microbial activity for wastewater treatment plant operation. Water Res. 246, 120737. doi: 10.1016/j.watres.2023.120737, PMID: 37857011

[B24] Priya K. Rani J. Gwal S. (2025). Chapter 6 - transforming agricultural residues to value-added products: waste to wealth. Sustain. Manage. Agro-food Waste, 69–85. doi: 10.1016/B978-0-443-23679-2.00006-9, PMID: 41735180

[B25] Quast C. Pruesse E. Yilmaz P. Gerken J. Schweer T. Yarza P. . (2013). The SILVA ribosomal RNA gene database project: improved data processing and web-based tools. Nucleic Acids Res. 41, D590–D596. doi: 10.1093/nar/gks1219, PMID: 23193283 PMC3531112

[B26] Rognes T. Flouri T. Nichols B. Quince C. Mahé F. (2016). VSEARCH: a versatile open source tool for metagenomics. PeerJ 4, e2584. doi: 10.7717/peerj.2584, PMID: 27781170 PMC5075697

[B27] Shinde R. Shahi D. K. Mahapatra P. Singh C. S. Naik S. K. Thombare N. . (2022). Management of crop residues with special reference to the on-farm utilization methods: a review. Ind. Crops Prod. 181, 114772. doi: 10.1016/j.indcrop.2022.114772, PMID: 41735180

[B28] Srinivas V. Naresh N. Pratyusha S. Ankati S. Govindaraj M. Gopalakrishnan S. (2022). Exploring plant growth-promoting streptomyces spp. for yield and nutrition traits in pearl millet hybrids. Crop Pasture Sci. 73, 484–493. doi: 10.1071/CP21438, PMID: 41161682

[B29] Sufyan A. Ahmad N. Shahzad F. Embaby M. G. AbuGhazaleh A. Khan N. A. (2022). Improving the nutritional value and digestibility of wheat straw, rice straw, and corn cob through solid state fermentation using different pleurotus species. J. Sci. Food Agric. 102, 2445–2453. doi: 10.1002/jsfa.11584, PMID: 34636045

[B30] Ukachi U. O. Rajasekar A. Gao B. Shen W. (2025). Dynamics and mitigation of antibiotic resistance genes during manure composting: A comprehensive review. Ecotoxicol. Environ. Saf. 304, 119152. doi: 10.1016/j.ecoenv.2025.119152, PMID: 41043234

[B31] van Rijssel S. Q. Kuipers E. Mason-Jones K. Koorneef G. J. van der Putten W. H. Veen G. F. (. (2025). Impact of soil inoculation on crop residue breakdown and carbon and nitrogen cycling in organically and conventionally managed agricultural soils. Appl. Soil Ecol. 205, 105760. doi: 10.1016/j.apsoil.2024.105760, PMID: 41735180

[B32] Wang Y. Chen Y. Wu W. (2021). Potassium and phosphorus transport and signaling in plants. J. Integr. Plant Biol. 63, 34–52. doi: 10.1111/jipb.13053, PMID: 33325114

[B33] Wei Z. Shen W. Feng K. Feng Y. He Z. Li Y. . (2022). Organic fertilizer potentiates the transfer of typical antibiotic resistance gene among special bacterial species. J. Hazard. Mater. 435, 128985. doi: 10.1016/j.jhazmat.2022.128985, PMID: 35483268

[B34] Włodarczyk K. Smolińska B. Majak I. (2022). Tomato allergy: the characterization of the selected allergens and antioxidants of tomato (solanum lycopersicum)—a review. Antioxidants 11, 644. doi: 10.3390/antiox11040644, PMID: 35453329 PMC9031248

[B35] Xing Y. Wang X. Mustafa A. (2025). Exploring the link between soil health and crop productivity. Ecotoxicol. Environ. Saf. 289, 117703. doi: 10.1016/j.ecoenv.2025.117703, PMID: 39808880

[B36] Zhang Y. Huang S. Cheng Y. Liu L. Li C. Zhang G. . (2025). Enhanced lignocellulose degradation and composts fertility of *flammulina filiformis* residue and eco-friendly insect (*protaetia brevitarsis)* composting by microbial agents-loaded biochar. Chem. Eng. J. 516, 164126. doi: 10.1016/j.cej.2025.164126, PMID: 41735180

[B37] Zhishen J. Mengcheng T. Jianming W. (1999). The determination of flavonoid contents in mulberry and their scavenging effects on superoxide radicals. Food Chem. 64, 555–559. doi: 10.1016/S0308-8146(98)00102-2, PMID: 41276264

