# Supplementary material for: Fungal-fermented corn straw as an organic amendment: balancing tomato nutrition, soil functions and antibiotic resistance
Source: Front Plant Sci. 2026 Feb 27;17:1765584. doi: 10.3389/fpls.2026.1765584 (PMC12982324; doi:10.3389/fpls.2026.1765584)
Supplement: Supplementary file 1 [file Supplementaryfile1.docx]

**Supplementary material**

**Fungal-Fermented Corn Straw as an Organic Amendment: Balancing Tomato Nutrition, Soil Functions and Antibiotic Resistance**

Table S1. Basic physicochemical properties of the fungal-fermented straw product (FSP) and the cultivation soil used in this study

| Parameter | Unit | FSP | soil |
| --- | --- | --- | --- |
|  |  | Value (mean ± SD) | |
| pH | – | 6.89±0.5 | 6.32±0.01 |
| EC | mS cm⁻¹ | 1.6±0.4 | 0.31±0.01 |
| TOC | g/kg | 373.85±29.65 | 14.01±0.57 |
| TN | g/kg | \|  \| 16.44±0.08 \| \| --- \| --- \| | 1.04±0.03 |
| C:N ratio | – | \|  \| 22.74±1.8 \| \| --- \| --- \| | 14.57±0.73 |

Notes: Values are presented as mean ± SD. For FSP, TOC, TN, and C/N ratio are expressed on a dry-weight basis. “–” indicates not applicable.

Table S2. Experimental design of FSP application methods and doses

| Code | Application Method | FSP Concentration | Depth | Notes |
| --- | --- | --- | --- | --- |
| WR | Control (No FSP) | 0% | **-** | No FSP added |
| DH | Deep application | 5% | 10 cm | High dose, deep application |
| DM | Deep application | 2% | 10 cm | Moderate dose, deep application |
| DS | Deep application | \|  \| 0.5% \| \| --- \| --- \| | 10 cm | Low dose, deep application |
| SM | Shallow application | \|  \| 2% \| \| --- \| --- \| | 3 cm | Moderate dose, shallow application |
| SH | Shallow application | 5% | 3 cm | High dose, shallow application |
| SS | Shallow application | 0.5% | 3 cm | Low dose, shallow application |


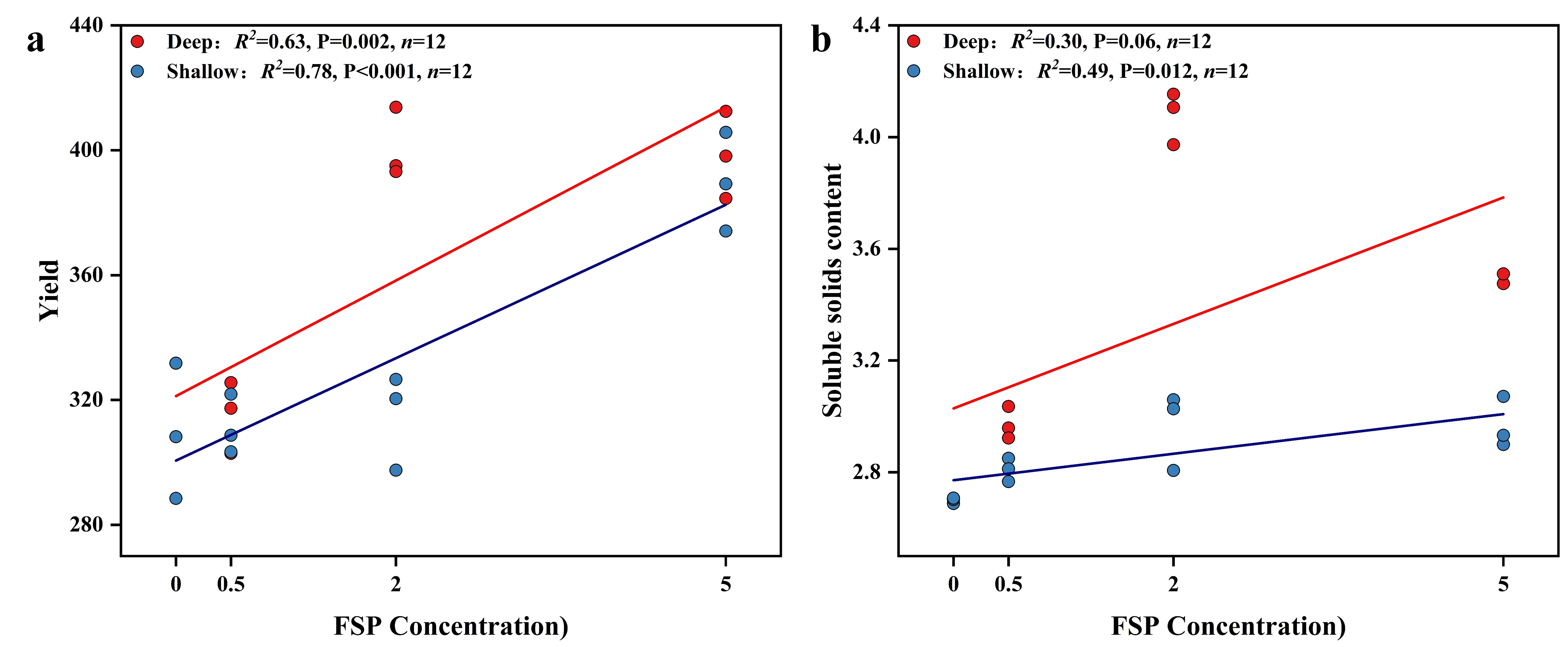


Figure S1. Dose–response relationships between FSP rate and yield/SSC under deep vs shallow incorporation.


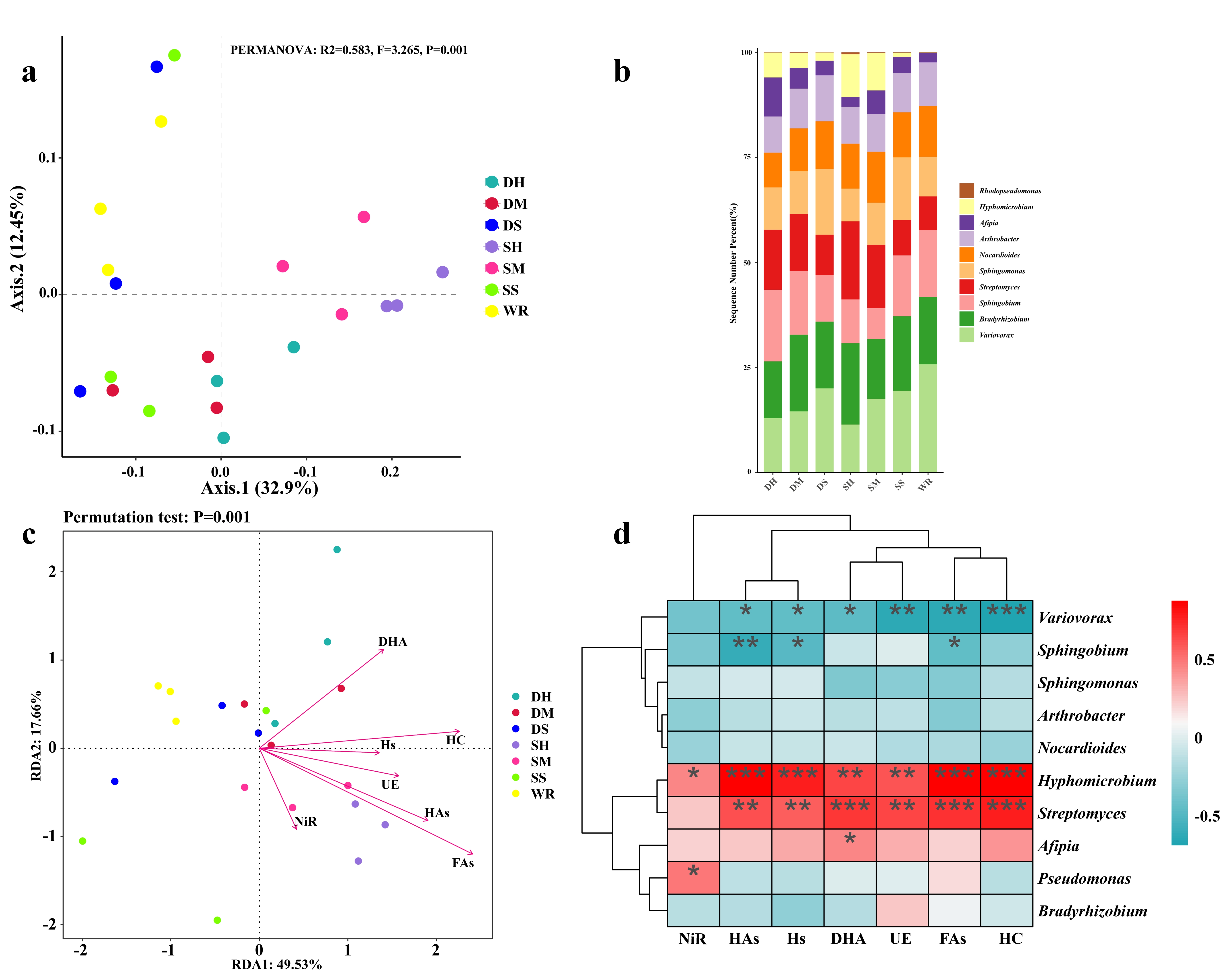
Figure S2. Bacterial community structure of tomato rhizosphere under FSP treatments and links to soil functions. (a) PCoA based on Bray–Curtis distances. Colors indicate treatments: DH (deep 5%), DM (deep 2%), DS (deep 0.5%), SH (shallow 5%), SM (shallow 2%), SS (shallow 0.5%), WR (without FSP). (b) Stacked bars of dominant bacterial genera (relative abundance, %). (c) Redundancy analysis (RDA) biplot showing associations between community composition and soil variables. (d) Heatmap of Pearson correlations between representative genera and soil variables. Color denotes correlation coefficient; * and ** indicate significance at P < 0.05 and P < 0.01, respectively.


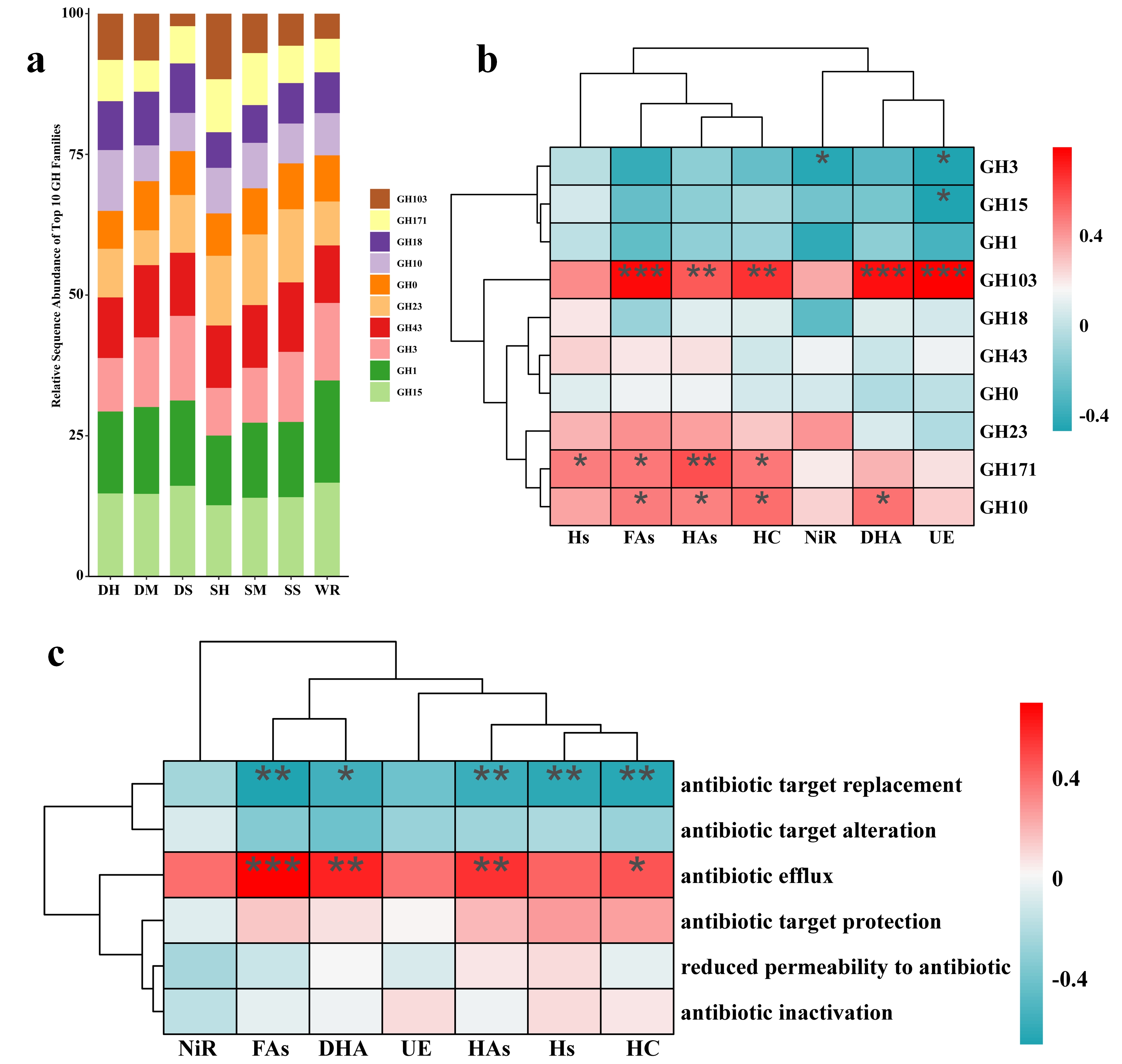
Figure S3. Glycoside hydrolase (GH) functional potential and antibiotic-resistance mechanisms in soils under FSP treatments. (a) Relative sequence abundance (%) of the top 10 GH families in metagenomes across treatments DH, DM, DS, SH, SM, SS, and WR. (b) Heatmap of Pearson correlations between GH-family abundances and soil variables. Colors denote correlation coefficients (red, positive; blue, negative); * and ** indicate significance at P < 0.05 and P < 0.01, respectively. (c) Heatmap of Pearson correlations between antibiotic resistance gene (ARG) categories—antibiotic target replacement, target alteration, antibiotic efflux, target protection, reduced permeability to antibiotics, and antibiotic inactivation—and the same soil variables. Colors and significance symbols as in panel (b).
